# Heck-type C-glycosylations in the synthesis of artificial nucleotides and functional nucleic acids

**DOI:** 10.1039/d6cb00078a

**Published:** 2026-03-26

**Authors:** Robert Dörrenhaus, Andre Zenz, Stephanie Kath-Schorr

**Affiliations:** a University of Cologne, Department of Chemistry and Biochemistry Greinstrasse 4 50939 Köln Germany skathsch@uni-koeln.de

## Abstract

Advances in nucleic acid chemistry have driven the development of a wide variety of artificial nucleobases. The synthesis of the glycosidic bond towards nucleosides still remains a major challenge in nucleic acid chemistry, particularly for C-glycosidic DNA nucleosides. This review highlights the Heck reaction as a central and versatile strategy for constructing diverse C-glycosidic DNA analogues. It discusses the underlying reaction mechanism, commonly employed catalyst and ligand systems and the scope of tolerated substituents. In addition, the review summarizes the subsequent applications of these artificial nucleobases with a particular emphasis on fluorescent nucleobase analogues.

## Abstract 2

Heck-reaction-mediated C-glycosylation enables efficient access to diverse artificial DNA nucleosides, supporting advanced studies of structure, function, and fluorescent nucleobase probes.

## Graphical abstract

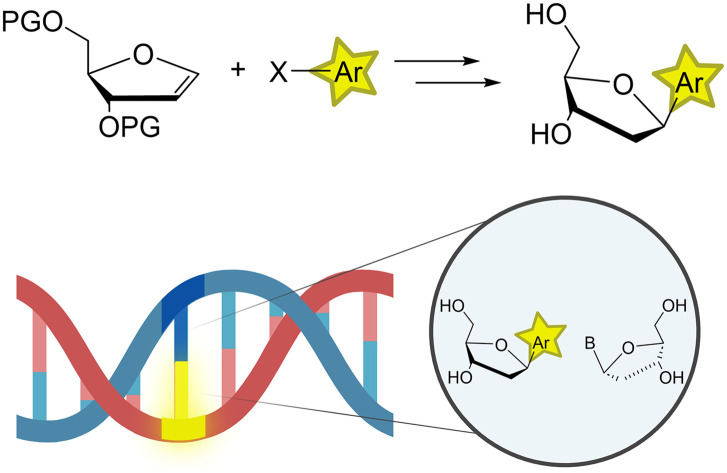

## Heck-type reactions towards artificial deoxyribose nucleic acids

Nucleic acids (NAs) are central to molecular biology and their identification fundamentally transformed our understanding of the chemical basis of life. Following their first isolation in the nineteenth century,^1^ decades of structural and biochemical investigations established DNA as the carrier of genetic information and revealed the molecular principles underlying heredity and replication.

**Scheme 1:**
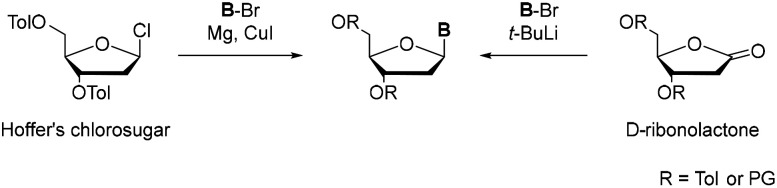
Methods towards C-glycosides starting from Hoffer's chlorosugar (left) and from protected D-ribonolactone (right).

These insights not only clarified the natural architecture of the genetic material but also inspired efforts to explore its chemical plasticity. Soon after the structural elucidation of DNA,^2^ researchers began to consider whether the canonical nucleobases were the only viable components of genetic systems. In this context, Rich proposed in 1962 that artificial base pairs could be incorporated into the genetic code, thereby opening the conceptual path toward an expanded genetic alphabet and synthetic nucleic acid systems.^3^ Since nature evolved the four lettered alphabet as an efficient compromise of high fidelity and information density, a third base pair would tremendously increase the information storage potential, expand the chemical space of nucleic acids and can be used to introduce functional groups into nucleic acids.^4–6^ It took more than 30 years from the idea to the development of the first unnatural base pair (UBP) and its successful incorporation into DNA.^7^ For the introduction of new artificial nucleobases into DNA and RNA, highly complex routes towards the desired nucleosides were developed. While nature solved the synthetic issue in a very elegant way *via* the enzymatic biosynthetic *de novo* pathway, researchers focused on classical synthetic routes. In biological systems nucleotides are assembled from simple metabolic precursors, including amino acids such as serine and glycine together with one carbon units through tightly regulated multi-enzyme pathways.^8–10^ In contrast, synthetic approaches typically begin from readily available small molecules that are elaborated into the desired nucleobases, which are subsequently coupled to ribose or deoxyribose scaffolds. The formation of N-glycosidic bonds has been achieved in a limited number of enzymatic systems,^11–13^ but most synthetic routes rely on classical chemical methodologies, particularly the silyl-Hilbert-Johnson reaction or Vorbrüggen-derived methods.^14–17^ Access to C-glycosidic nucleosides has proven considerably more demanding. Extensive methodological studies have therefore focused on improving C-glycoside formation, motivated by their value in nucleobase design and by their enhanced resistance to enzymatic and acid-mediated cleavage relative to N-glycosidic analogues.^18^ The formation of a C–C bond between a ribose scaffold and a nucleobase remains a central synthetic challenge in the development of new unnatural base pairs. The difficulty arises from the steric demand and electronic deactivation of the highly substituted coupling partners, as most strategies require the construction of a bond between an sp^2^ hybridised carbon of the heteroaromatic nucleobase and the anomeric sp^3^ hybridised carbon of the sugar moiety. Despite these constraints, several C-glycosidic nucleoside analogues have been successfully realized and broadly implemented in expanded genetic systems.^19–21^ These nucleosides were prepared using a long-established route involving aryl lithium substrates and sugar lactones (Scheme 1).^22^ The protected D-ribonolactone derivatives serve as modular ribose electrophiles which undergo nucleophilic addition with lithiated C-nucleophiles to give the corresponding hemiketal intermediate, which is subsequently reduced by a silane in the presence of a Lewis acid *via* hydride transfer. The β-anomer is the predominant product as the reduction selectively occurs from the α-face due to the kinetic anomeric effect.^23^ Even though this method is applicable in large-scale synthesis, it offers low yields and excludes many electrophilic and acidic functional groups at the nucleobase.^24,25^

Complementary to these lactone-based approaches, another pre-Heck strategy for C-nucleosides attributed to Hoffer who first described the Hoffer's chlorosugar (1-chloro-2-deoxy-3,5-di-O-toluoyl-α-D-ribofuranose) was described.^26^ This sugar moiety is a versatile 2’-deoxyribosyl donor that undergoes nucleophilic substitution at C1’ with aryl-organometallic reagents, such as organocopper species to yield C-nucleosides.^27^ In parallel, electron rich arenes can be directly C-glycosylated by Lewis acid-promoted Friedel–Crafts type alkylations using activated ribose donors such as 1-O-methyl-3,5-di-O-toluoyl-2-deoxyribose, providing concise access to aryl-C-nucleosides without pre-functionalized organometallics.^28^ Leumann and co-workers have employed both classes of sugar building blocks in their work, using Hoffer-type 2′-deoxyribosyl donors and ribonolactone-based strategies to assemble conformationally constrained and heterocyclic C-nucleosides, which later served as platforms for more recent applications such as fluorescent or quenching C-nucleoside probes.^29,30^ The stereocontrol of these C-nucleosides is achieved *via* neighbouring group participation. For Hoffer's chlorosugar, the toluoyl groups aid to favour β-configuration, while for the lactone, also other large protecting groups can be employed. Nevertheless, α/β-selectivity was mostly described between 1 : 2–1 : 3.^28,29^ The moderate yields, comparatively low β-selectivity and the limitations with respect to several aryl electrophiles demonstrated the demand for a more general pathway for C-glycosidation.

Consequently, cross-coupling reactions, a mature and versatile class of transformations widely employed in both industrial and academic settings, were introduced into nucleoside synthesis. Substantial efforts have been devoted to the refinement of catalysts and precatalysts, as well as to broadening the substrate scope toward a wider range of electrophilic partners.^31–34^ C-glycosidic bond formation at ribose scaffolds is well established and encompasses a broad spectrum of synthetic approaches.^35–37^ In contrast, this review concentrates on C-glycosidic bond formation at deoxyribose moieties as a strategy to access novel unnatural nucleotides for incorporation into DNA. Among the available methods, the Heck reaction has emerged as the predominant transformation for constructing the requisite carbon–carbon linkage.

## General mechanism of the Heck reaction with furanoid glycals

Numerous cross-coupling strategies have been developed for nucleoside modification. For example, Ni-catalysed cross-coupling *via* furanosyl radicals has proven highly effective for RNA nucleosides.^37,38^ These transformations typically proceed in high yield and with pronounced β-selectivity. The stereochemical outcome has been attributed to the substituent at the C2′ position, whose protection imposes steric demand that disfavours the undesired α-attack. In the context of DNA nucleoside synthesis, however, such approaches are less advantageous. The absence of a substituent at the C2′ position eliminates this steric bias, thereby reducing intrinsic stereocontrol and complicating β-selective C-glycosidic bond formation. While Heck reactions for glycosylations are well described,^33^ the review published by Benner in 2006 remains the most comprehensive summary of Heck reactions leading to DNA C-glycosides to date.^39^

The pioneering work in Heck-type reactions forming C-glycosidic bonds with the anomeric carbon was reported by Daves and co-workers in several publications starting from 1978.^40–42^

**Scheme 2:**
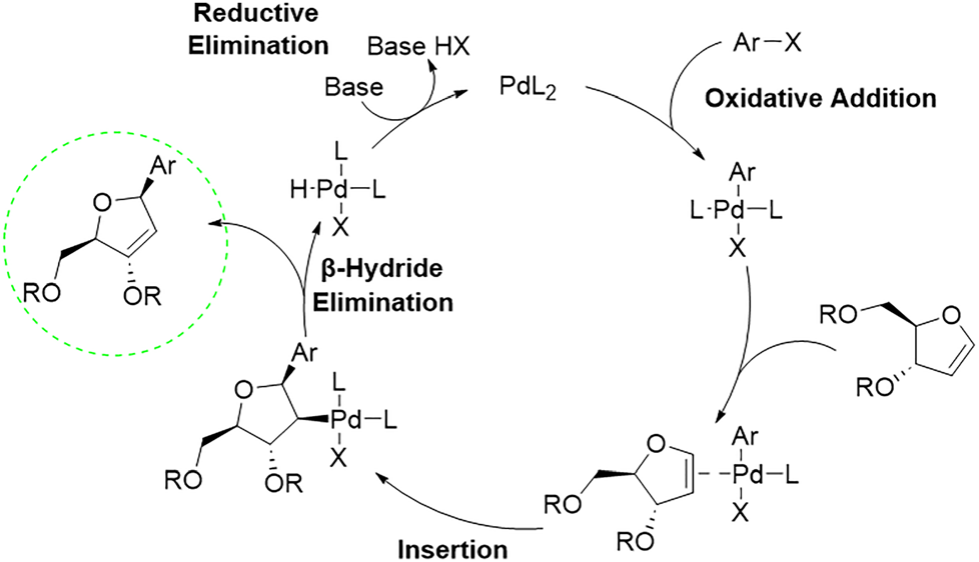
Schematic palladium-catalysed Heck reaction of an arylhalogenide with a glycal.^43^

The general Heck reaction, named after Richard F. Heck was first reported in the 1960s through the palladium catalysed coupling of arylmercury compounds with olefins.^43^ The catalytic cycle (Scheme 2) begins with a palladium (0) species, which forms a palladium(ii) complex after oxidative addition of an arylhalogenide. Subsequent coordination of the alkene, for example a glycal, enables migratory insertion into the aryl palladium bond in a *syn*-selective manner. A β-hydride elimination takes place, which forms the desired coupling product and a palladium(ii) complex. Finally, base assisted elimination of HX regenerates the palladium (0) catalyst, thereby closing the catalytic cycle.

**Scheme 3:**
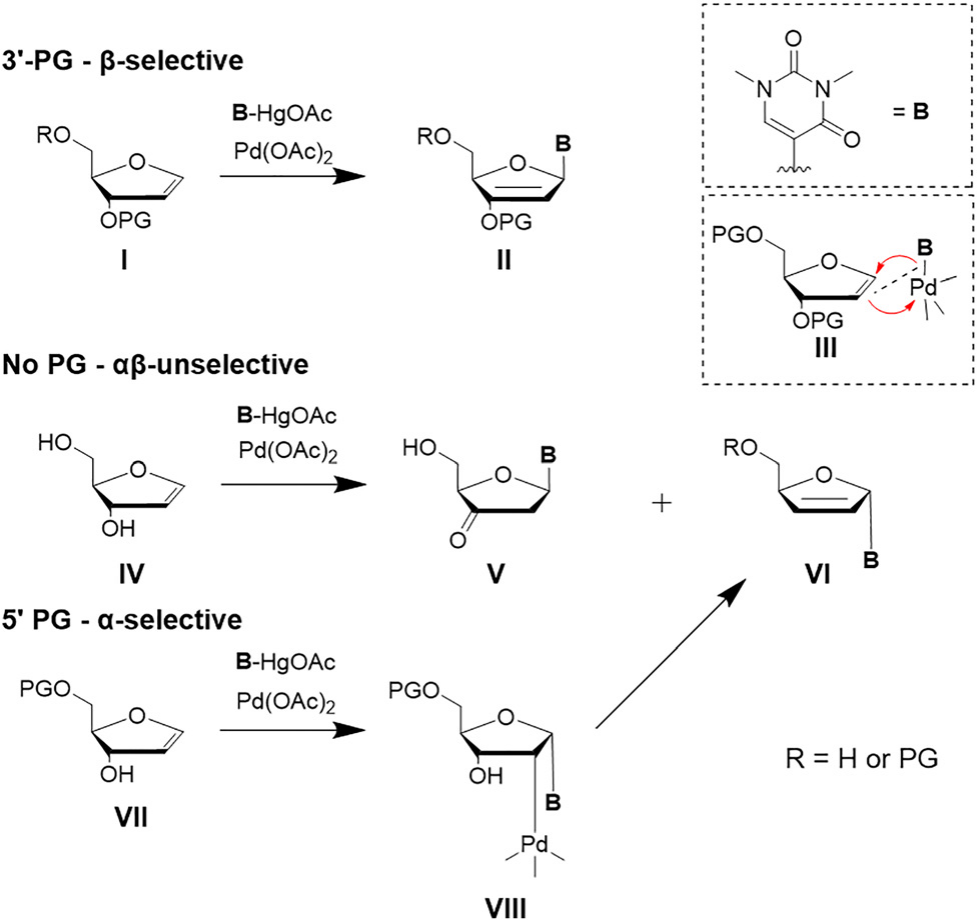
Overview of the influence of protecting groups on the stereochemistry of a palladium(ii)-mediated Heck reaction with furanoid glycals. If the 3′ or both hydroxy groups are protected, β-selectivity is observed. If only the 5′ OH is protected, α-selectivity is predominant. No protecting groups lead to an unselective outcome.^41^

It was demonstrated that Heck reactions can proceed regioselectively and stereoselectively in the C–C bond formation for C-glycosides under suitable conditions.^43,44^ The main driving force for stereoselectivity in these reactions is, analogous to related reactions towards RNA nucleosides, the protecting group strategy. In particular, a protecting group at the C3′ hydroxy group of the furanoid glycal controls the anomeric configuration of the C-glycosidic product. The glycosidic bond forms at the least sterically hindered face of the ring system of the glycal during the attack of the organopalladium reagent (Scheme 3).^41^ With a protecting group at the C3′ hydroxy function, a selective formation of the β-anomer can be observed.

The key step of the catalytic cycle is the *syn*-migratory insertion step III, which for furanoid glycals proceeds from the less sterically hindered face of the substrate. This facial selectivity determines the anomeric outcome of the C-glycosidic product. After insertion, β-hydride elimination takes place generating a Pd-H bond and a palladium-alkene π-complex. Subsequent dissociation of the alkene restores the Pd(ii)-species. Finally, base assisted reductive elimination regenerates the Pd(0) catalyst. If the furanoid glycal is protected at the C3′ (Scheme 3, I), the *syn*-addition occurs from the β-face (III). *Syn*-Elimination involving the proton of the C3’ carbon yields the protected enol (II).^45^ In contrast, when only the C5’ hydroxy group is protected (Scheme 3, VII), *syn*-insertion of the Pd(ii)-complex proceeds from the α-face (VIII). In this case, the intermediates lacking a *syn* β-hydrogen group undergo *syn* palladium-oxide elimination towards VI.^46^ In the absence of any protecting group (Scheme 3, IV), stereocontrol is significantly diminished and only low selectivity is observed with a ratio of α : β = 1.6 : 1 affording a mixture of V and VI. Although introduced decades ago, this transformation continues to exert substantial influence on contemporary strategies for the synthesis of C-glycosides. Glycal donors are readily accessible, and the Heck reaction exhibits broad functional group tolerance, accommodating a wide range of substituted coupling partners. In early works, Daves and co-workers used mercurial derivatives as the aryl RX component. These were subsequently replaced by more sustainable electrophiles, most notably aryl halides, as reaction conditions were refined and optimised.^39^ Additionally, a highly stereoselective reduction pathway for the 2′-deoxy-3′-keto C-nucleosides towards the DNA nucleosides using mild and sterically demanding reducing agents such as sodium triacetoxyborohydride was established.^47^ Heck-type methodologies were further implemented in the synthesis of components of unnatural base pairs, such as of dxT by Kool^48,49^ and of MMO2 (Fig. 7) and other substituted benzene analogues by Romesberg.^50^ Very recent work in the synthesis of unnatural nucleosides was performed by Wagenknecht and co-workers to develop new C-glycosides in excellent yields.^51,52^ In these studies, further optimisations were taken into account, including different Pd-ligand complexes. However, the same principle and furanoid glycals and reducing methods as described previously by Daves were used, highlighting the actuality and relevance of this Heck-type reaction for DNA nucleoside development.

## Recent synthetic application of the Heck reaction towards different DNA substrates

The furanoid glycal constitutes a central substrate class in the application of the Heck reaction for the synthesis of DNA nucleosides. Glycals can be found in literature since their first mention in 1913.^53^ Furanoid glycals in particular can be traced back to Fletcher and co-workers, who first published a benzyl protected glycal, synthesized in three steps from protected ribose.^54^ This strategy remained widely used until Pedersen introduced a more direct route to furanoid glycals.

**Scheme 4:**
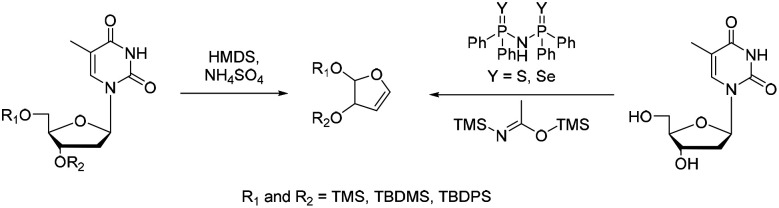
Synthesis of the furanoid glycal.

In this approach, thymidine is subjected to treatment with HMDS in the presence of ammonium sulfate, effecting elimination of the nucleobase and formation of the corresponding glycal (Scheme 4).^55^ Herein different protecting groups at 3′-OH and 5′-OH influence the glycal formation yield: In general silyl ethers are preferred, with bulkier TBDPS-groups at 5′ and/or 3′ leading to good yields.^56^ This approach is still one of the most commonly used to form furanoid glycals. Subsequent refinements have further enhanced its practicality. For example, by Mao *et al.*, who identified a series of organocatalysts that efficiently form the desired glycals from nucleosides and BSA under milder conditions.^57^ Depending on the protecting-group strategy, furanoid glycals can be bench stable and are easily available in large-scale synthesis, which makes them valuable precursors for UBP synthesis.^58^

**Fig. 1:**
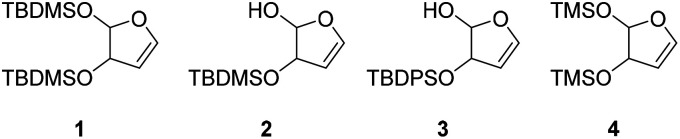
Glycals for reactions described in Table 1.

Broader interest in Heck reactions involving sugar moieties emerged alongside the development of artificial base pairs. In 1978, Daves demonstrated that a dihydropyran substrate, when subjected to Pd(OAc)_2_ (1 eq.) and LiCl (1 eq.), affords a product with well-defined stereochemistry and conformation.^40^ The first publication using furanoid glycals was published in 1983^59^ and optimised in 1986, when Daves and co-workers used uracil mercury acetate (Scheme 3, B) and 1 eq. Pd(OAc)_2_ with furanoid glycals.^41^ Later, in 1992, Daves also optimised the reaction conditions by using a 3′-TBDPS protected furanoid glycal, changing the mercury acetate to iodouracil and reducing the catalyst loading to 0.1 eq. Pd(OAc)_2_ and 0.2 eq. AsPh_3_ to yield 63% of the nucleoside after reduction. Since these initial reports, a wide range of substrates and reaction conditions has been explored, further elaborating on the Heck-type reaction (Table 1 and Fig. 1).^47^

**Table 1:** Selected DNA C-nucleosides, synthesized *via* Heck reaction, encompassing diverse catalyst/ligand systems, tolerated substituents, and isolated yields.^18,47,50,51,60–68^ Used glycals were shown in Fig. 1

Glycal	Electrophile/yield	Catalyst	Substituents^a^
2	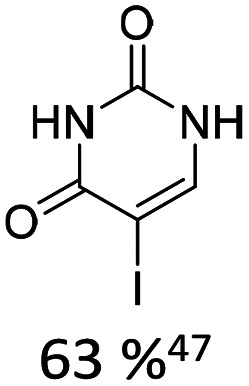	10% Pd(OAc)_2_, 20% AsPh_3_	ketones, hetero N
1	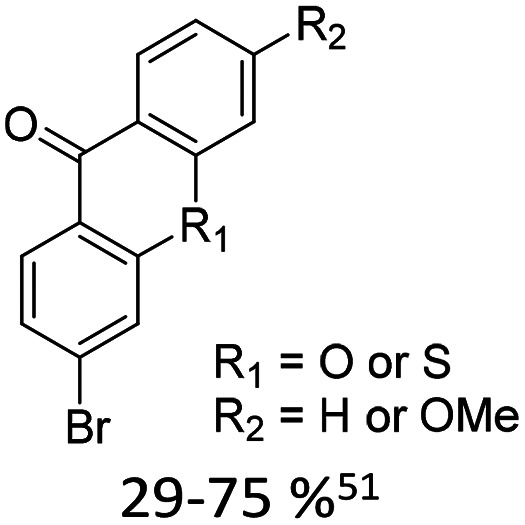	15% Pd(dppf)Cl_2_ or 5% BrettPhos Pd G3	bi- and tricycles, no substituents on the first benzo ring
2	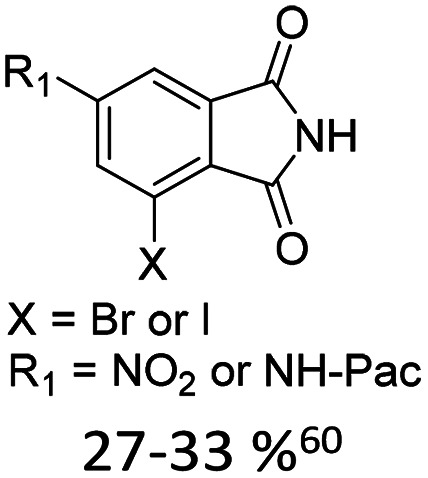	20% Pd(OAc)_2_, 40% P(PhF_5_)_3_	NO_2_, NHR
2	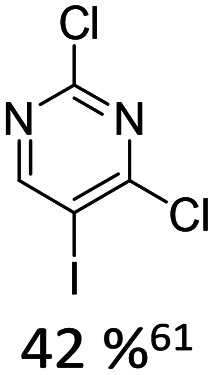	20% Pd(OAc)_2_, 40% P(PhF_5_)_3_	Cl, hetero N
1 or 2	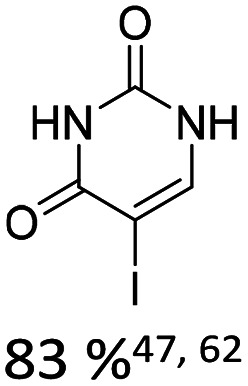	10% Pd_3_dba_2_, 20% XantPhos	ketones, hetero N
1	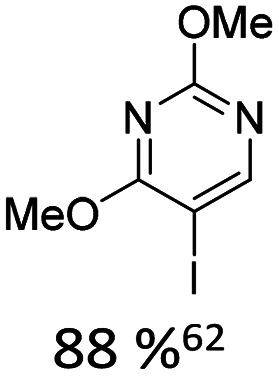	10% Pd(OAc)_2_, 20% PPh_3_	OMe, hetero N
4	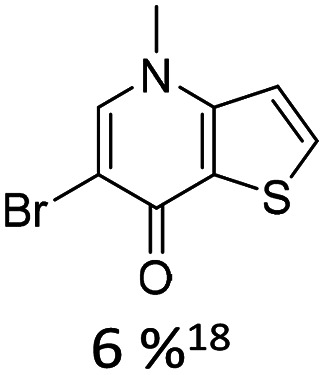	15% Pd(dppf)Cl_2_	ketone, NR_3_
1 or 2	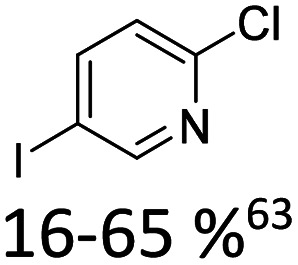	20% Pd(OAc)_2_, 40% AsPh_3_	Cl, hetero N
1	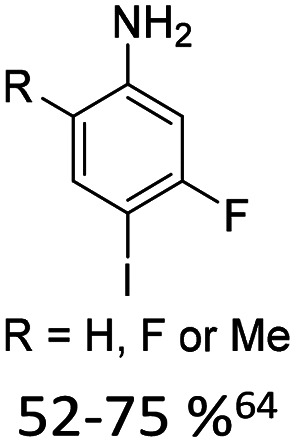	15% Pd(OAc)_2_, 45% AsPh_3_	NH_2_, alkyl, F
2	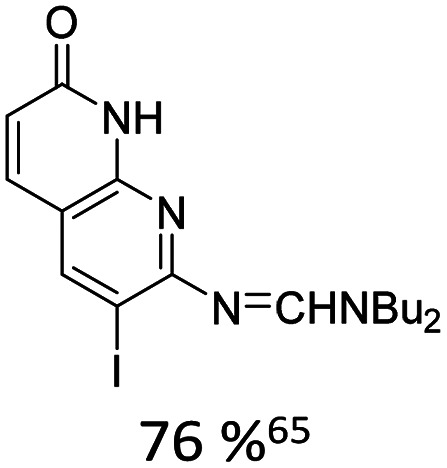	10% Pd(OAc)_2_, 20% AsPh_3_	NR_2_
2	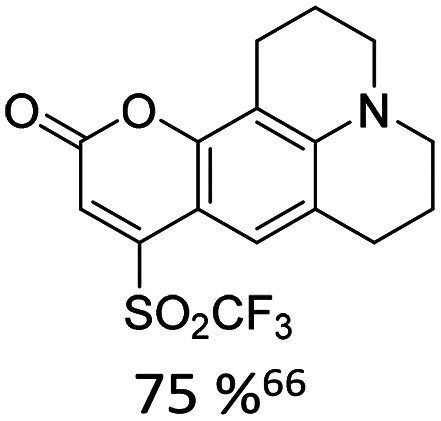	40% Pd(OAc)_2_, 5% bppp	ketone, hetero O
3	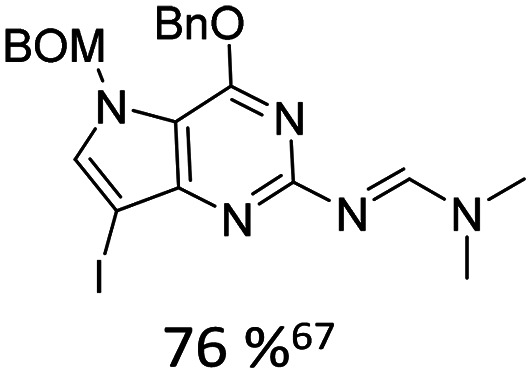	47.5% Pd(OAc)_2_, 475% *n*Bu_4_NCl	protected hetero N
1	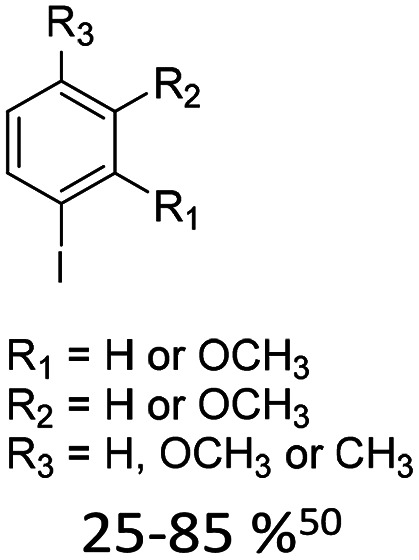	10% Pd(OAc)_2_, 20% AsPh_3_	CH_3_, OCH_3_
1	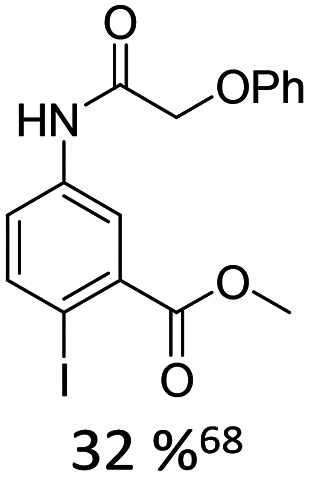	12% Pd(OAc)_2_, 26% AsPh_3_	COOMe, NHR

a Only on the first ring. Substituents further away in multicyclic nucleobases were omitted.

**Fig. 2:**
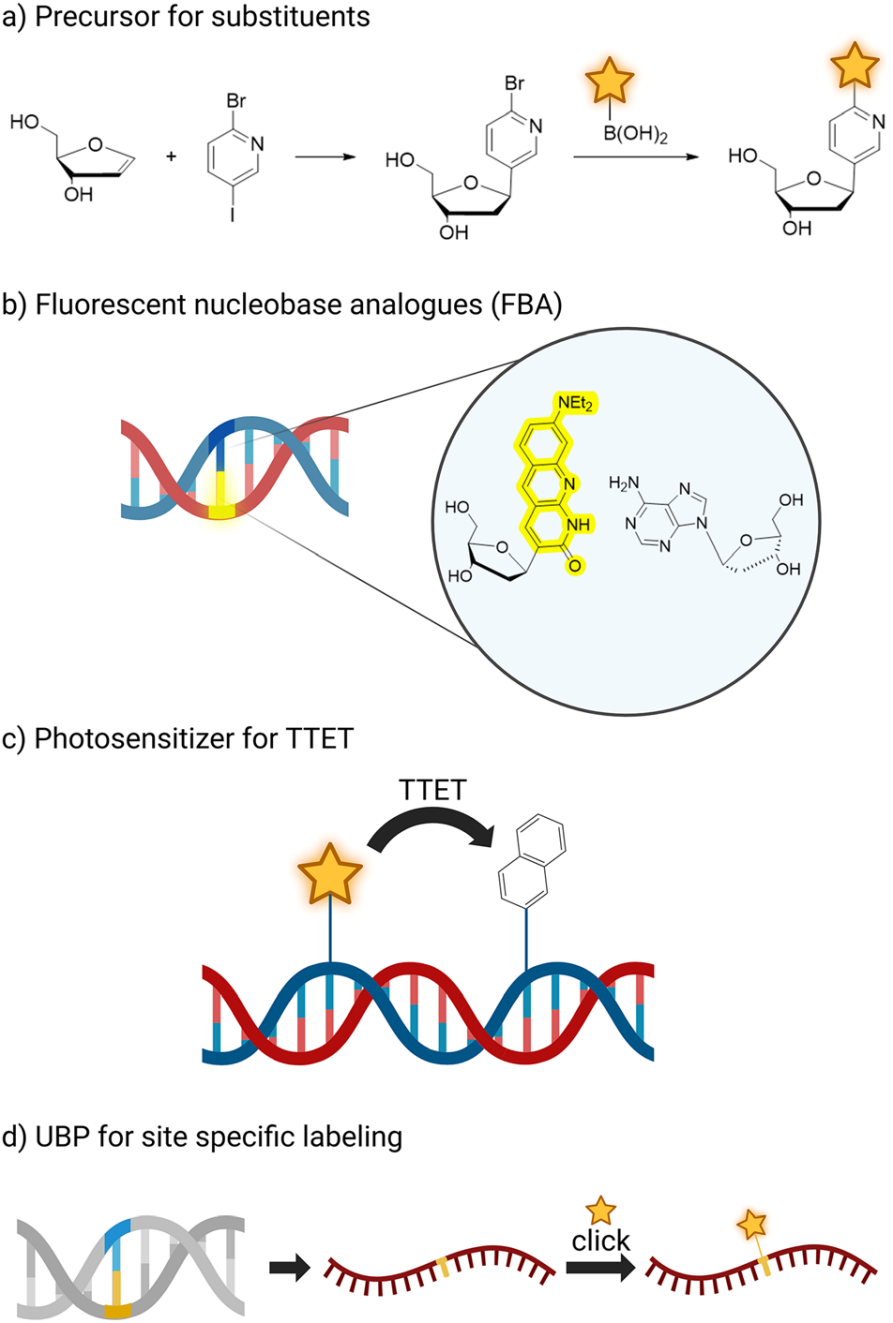
Schematic overview of different applications of C-glycosides synthesized *via* Heck reaction. (a) Precursor for further substituents, *i.e.* attachment of a fluorophore after glycosidation; (b) fluorescent nucleobase analogues – unnatural nucleobases used as fluorophore themselves; (c) photosensitizer for triplet–triplet-energy-transfer within a DNA strand; (d) unnatural base pairs for site specific labelling in RNA. (Created in BioRender. Kath-Schorr, S. (2026) https://BioRender.com/llljfrd).

As summarized in Table 1, Heck reactions involving glycals rely exclusively on palladium centered catalysts, while Pd(OAc)_2_ and AsPh_3_ remains one of the most significant catalyst-ligand systems. Tolerated arylhalogenides are bromides and iodides. Early examples such as pseudouridine, first accessed through glycal coupling by Daves, illustrate the notable functional group tolerance of this methodology. Heteroatoms, ketones, and enol ethers are compatible with the reaction conditions. In addition, substrates bearing halogens, alkyl substituents, amines and imines have been successfully employed. Even bi, tri, and polycyclic aromatic systems do not significantly compromise reactivity. A particularly noteworthy trend emerges when comparing C-glycosidic DNA nucleoside synthesis *via* glycals with related cross coupling approaches to ribose derivatives in RNA chemistry. In ribose based systems, electron withdrawing substituents such as ketones positioned proximal to the coupling site are generally considered deactivating and can impede the reaction.^35,38^ In contrast, for Heck reactions with glycals, electron withdrawing groups such as ketones often exert a beneficial effect, whereas electron donating substituents, for example amines, may reduce efficiency and frequently require protection. This observation aligns with the general principle that electron deficient aryl halides typically undergo oxidative addition more readily in Heck reactions than electron rich counterparts.^69^ Furthermore, aryl halides featuring a second halogen, were often introduced for post-coupling modification (Fig. 2a). This allowed an even higher substrate scope, as this sets the limit of possible substituents to the scope of the follow up reaction, which could be *i.e.* a Suzuki coupling.^70^

Post-Heck reaction modification plays an important role for the substrate scope. Several applications were achieved by late-stage derivatisation of the C-glycosidic Heck-products, for example intrinsically fluorescent nucleobases were modified after Heck-coupling to implement unfavoured substituents.

In summary, the Heck reaction constitutes a powerful strategy for the synthesis of C-nucleosides and has proven particularly effective for the incorporation of electron deficient heteroaryl partners. Post-coupling derivatization further expands structural diversity and enables access to substitution patterns that would be difficult to introduce directly. The stereochemical outcome can be easily adjusted by the protecting group strategy. Despite its synthetic utility, the reported examples largely focus on individual nucleoside targets rather than systematically exploring a broad substrate scope. Most reactions employ the Pd(OAc)_2_/AsPh_3_ catalyst/ligand system, while some derivatives require more specific combinations (Table 1). The implementation of Heck chemistry with furanoid glycals has enabled the development of numerous structurally novel C-nucleosides. Selected recent applications of these compounds will be discussed in the following sections.

### Fluorescent nucleosides

Fluorescent labels underpin a wide range of technologies for the detection and quantification of nucleic acids. A broad repertoire of fluorophores is available, and multiple attachment sites within nucleic acid architectures have been explored.^63^ Fluorescent labelling can be introduced by attachment of fluorophores at different sites like 2′-C of deoxyribose, 5′-OH of oligonucleotide or C5 of pyrimidines. In addition, labelling strategies have been implemented through functionalisation of artificial base pairs, enabling post-transcriptional modification *via* click chemistry^71^ (Fig. 2d) or an intrinsically fluorescent nucleobase analogue as illustrated in Fig. 2b. Such fluorescent base analogues (FBAs) often provide sensitive probes for base pairing, stacking interactions, and local conformational dynamics within nucleic acids. While numerous examples of fluorescent N-glycosidic nucleosides have been reported, the following section focuses specifically on fluorescent C-glycosidic nucleosides accessed through Heck-type methodologies.

Many studies have examined oligonucleotides containing C-nucleotides, most of which were prepared by phosphoramidite chemistry on solid support. Only a limited number of such modified nucleosides, including MMO2, CTPT3, and 7–8, have also been shown to be compatible with enzymatic incorporation as nucleoside triphosphates.

Fluorescent probes derived from C-nucleosides enable sensitive detection of oligonucleotides. Several systems display pronounced changes in emission intensity or wavelength as a function of their microenvironment, including hybridisation state and the identity of the opposing base. These environmentally responsive properties expand their utility beyond simple labelling, allowing interrogation of base pairing fidelity and local structural dynamics.

**Fig. 3:**
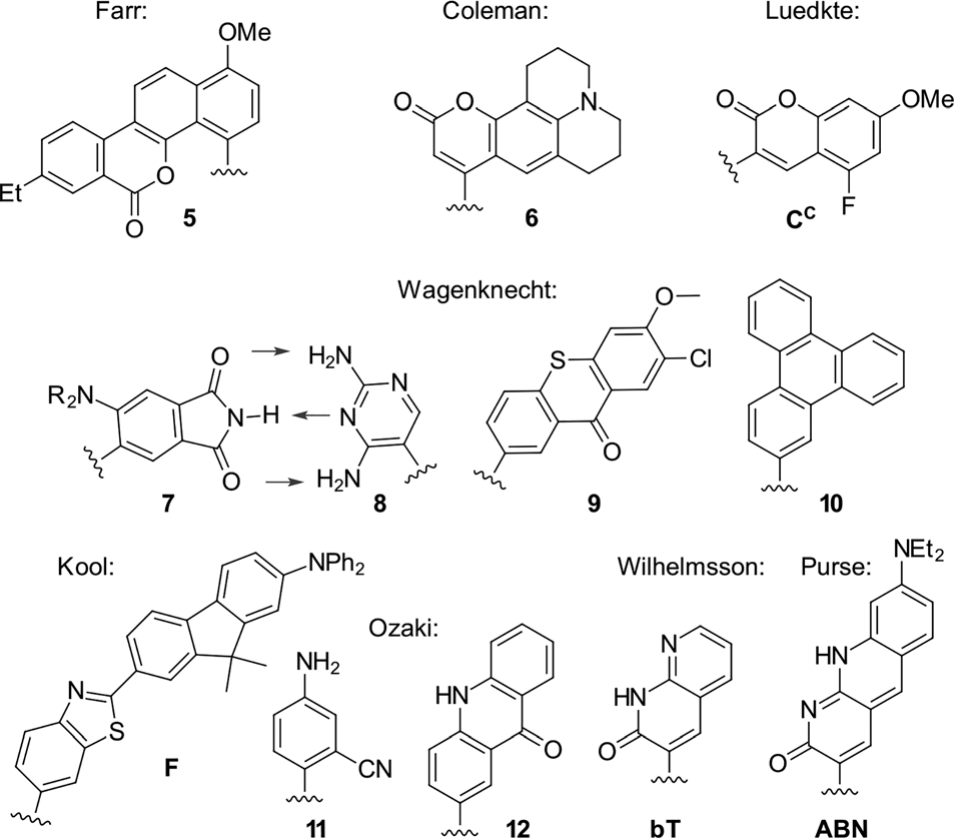
Fluorescent C-nucleosides, including hydrogen-bond base pairing.

When appropriately designed with respect to size, geometry, and hydrogen bonding capability, C-nucleosides can function as unnatural base pairs while exerting only minimal perturbation on the overall duplex architecture. For example, Coleman reported the synthesis of a coumarin C-nucleoside 6 (Fig. 3),^66^ which was later incorporated into dsDNA opposite to an abasic site.^72^ Spectroscopic analysis during duplex melting experiments indicated that the fluorophore intercalates within the helical stack between adjacent natural base pairs.

Distinct shifts in both absorption and emission maxima were observed when comparing single stranded and double stranded forms, consistent with pronounced sensitivity to the local microenvironment in each structural state. The synthesis of related coumarin C-nucleoside analogue, C^C^, which can be incorporated enzymatically opposite to guanine was described by Luedtke.^73^

Using the Heck-coupling strategy, Ozaki and co-workers synthesized a fluorescent aminobenzonitrile 11,^74^ as well as an aminobenzoic acid nucleobase analogue. These compounds displayed pronounced sensitivity of their emission properties to variations in solvent composition and pH, highlighting their potential as environmentally responsive probes within nucleic acid frameworks.^68^ In addition, a patent granted in 2010 to members of the same research group disclosed related fluorescent nucleoside analogues featuring an acridone core (12).^75^ These developments further underscore the versatility of Heck derived C-nucleosides as platforms for the integration of photophysically active heterocycles into DNA architectures.

Kool developed a structurally expanded green-emitting benzothiazol-fluorenyl derived C-nucleosidic fluorophore, F (Fig. 3). This chromophore was subsequently applied in protein tagging strategies, demonstrating that C-glycosidic fluorescent nucleosides can serve not only as reporters of nucleic acid structure but also as versatile tools in biomolecular labelling.^76^

The Wilhelmsson and Grøtli research groups have synthesized a bicyclic thymidine analogue, bT, *via* Heck-coupling and investigated its photophysical behaviour within oligonucleotide contexts.^77^ Building on this concept of expanded aromatic nucleobases Purse and co-workers introduced the larger fluorescent ABN nucleobase analogue which retains the ability to pair with adenine as illustrated in Fig. 3.^78^ In a subsequent study, the same groups demonstrated that nucleic acids containing single ABN nucleotides can be detected *via* total internal reflection fluorescence microscopy (TIRFM) enabling single-molecule-level observation. Detailed photophysical analyses clarified the origin of its favourable emission properties, including quantum yield and environmental responsiveness within duplex DNA.^79^ They also revisited and optimised their original Heck-coupling synthesis. The combination of replacing the bromo- by the corresponding iodo-aryl and employing the 3′-TBDPS-5′-OH-glycal 2 instead of the 3′,5′-bis-TBS-glycal 1 further improved the reactivity. Together, these modifications veritably improved coupling yield from 20% to 40%.

### Photosensitizing base analogues

The Wagenknecht group employed the Heck reaction to access C-glycosidic nucleoside analogues that are not intrinsically fluorescent, but interact with light by acting as photosensitizers in mechanistic studies of triplet energy transfer. Upon sensitization, these systems can induce cyclobutane formation between adjacent pyrimidine bases, a well-established pathway of DNA photodamage that occurs naturally upon UV irradiation and is associated with the development of skin cancer.^51^ Initially, triphenylene (10) and xanthone as well as thioxanthone derived motifs (9) were incorporated into C-nucleosides. Subsequent work expanded this platform through the introduction of structurally diverse xanthone derivatives, enabling systematic modulation of triplet energy levels.^80^ By fine tuning the photophysical properties of these sensitizers, the group was able to probe the triplet energy landscape governing pyrimidine dimer formation with enhanced precision. The photosensitizer C-nucleotides were incorporated into defined oligonucleotide sequences in which the chromophore was positioned at distances ranging from zero to three base pairs relative to adjacent thymine residues. Upon irradiation, analysis of the reaction products demonstrated that the photochemical dimerization rate of thymine decreased with increasing spatial separation from the sensitizer, indicating an inverse relationship between distance and cyclobutane formation efficiency. Furthermore, the triplet energy gap of different sensitizers correlates with the cyclisation rate of the T-dimers. Similarly, Wagenknecht also introduced 4-substituted benzophenones as photosensitizers *via* Heck reaction to generate the respective C-nucleosides. Selected derivatives were shown to catalyse an intramolecular [2+2] photocycloaddition of coumarins. The authors have implemented those also in the binding pocket of an aptamer binding the coumarin substrate, which led to a preferential stereochemical outcome of the photocycloaddition^81^ and they envision that such nucleoside analogues could be adapted to work in other photocatalytically active DNAzymes in the future.^82^

In a separate study, the Wagenknecht group developed an C-glycosidic unnatural fluorescent base pair consisting of (dimethyl-)amino-phthalimide (7) and a diaminopyridine (8) base analogue.^52^ The design combined hydrogen bonding complementarity with intrinsic fluorescence of the phthalimide chromophore. Biochemical experiments showed that this unnatural pair can be incorporated and further extended by a DNA polymerase, exemplifying compatibility with enzymatic replication. The pair was further characterised by extensive structural validation by NMR spectroscopy, which confirmed the formation of a defined base pair within the duplex, supporting the proposed pairing geometry and demonstrating that C-glycosidic fluorescent analogues can participate in stable and replicable artificial base pairing systems. Building on these structures, the group subsequently examined the fluorescence properties of duplexes in which the phthalimide nucleobase analogue 7 was positioned opposite different canonical nucleotides. Distinct shifts in emission maxima were observed in relation to the opposing base, demonstrating that the fluorophore responds sensitively to its pairing partner. In addition, fluorescence was found to be markedly enhanced in double stranded constructs relative to the corresponding single strands, rendering 7 a promising non-specific turn-on fluorescence probe for hybridisation events.^83^ The base pair was further investigated by computational approaches, including molecular dynamics simulations of duplex structures and calculations of photophysical properties.^84,85^

The phthalimide nucleoside analogue isomer attached at C6 of the aromatic ring (see Table 1, entry 3), when paired with 8, exhibited an improved stacking geometry, showing a near-native B-DNA structure.^86^

### C-nucleosides with advanced functionalities

**Fig. 4:**
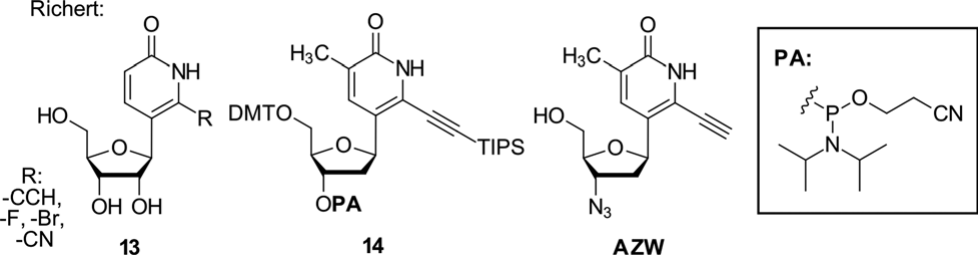
Strongly adenine-pairing nucleosides reported by Richert, including ribonucleoside, phosphoramidite reagent for solid phase synthesis, and 3′-azide.

Recently Richert and co-workers have transferred the Heck-coupling strategy also to the synthesis of ribonucleosides (13).^87^ The transformation proceeds through oxidation of the 3′-silyl-enol ethers to 2′-hydroxy-3′-ketones with trifluorodioxirane (TFDO) followed by reduction of the ketone to yield the ribonucleoside. This sequence provides a synthetically divergent entry to both 2′-deoxy and 2′-hydroxy nucleosides from common intermediates, which is particularly attractive in contexts where parallel evaluation of DNA and RNA analogues is desired. An analogous route was also followed to access the 3'-azide derivative AZW as shown in Fig. 4. The resulting nucleoside series includes derivatives bearing bromo, fluoro, cyano, and ethynyl substituents. In particular, the ethynyl and cyano variants were designed to enhance pairing preference for adenine over thymine. Such strengthened adenine pairing had previously been demonstrated for the corresponding deoxyribonucleoside analogue prepared *via* Heck-coupling, underscoring the modularity of this synthetic platform in tuning base pairing properties.^88^

**Fig. 5:**
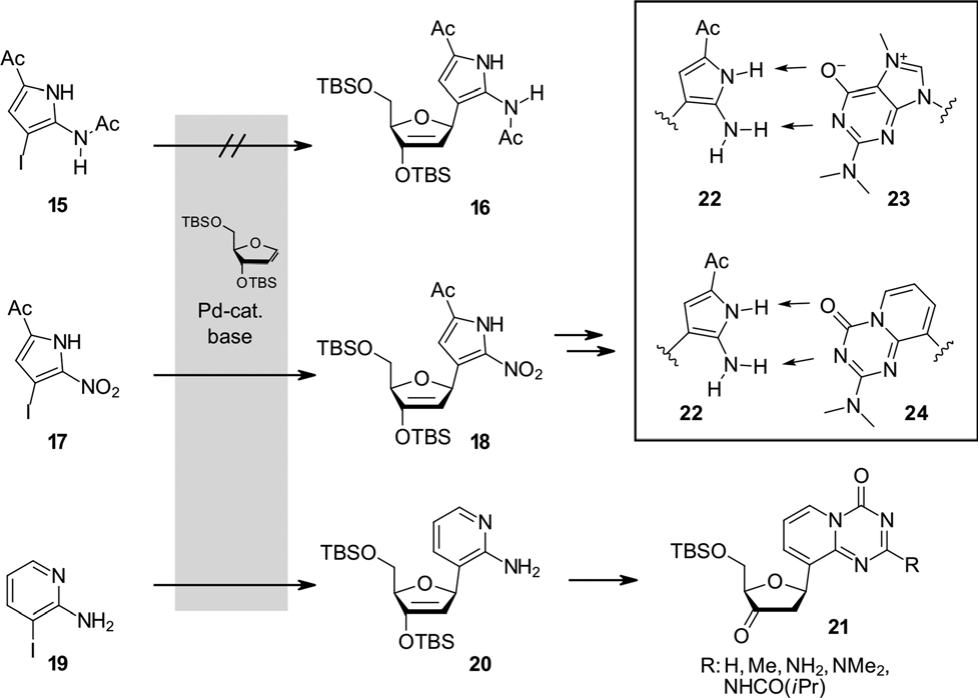
Example of electronic influence of the aglycon on coupling efficiency: the electron-poorer aglycon 17 couples readily in contrast to 15 for the synthesis of the pyrrole nucleobase analogue pairing to either trimethyl guanosine or an unnatural C-glycosidic triazine base analogue.

Oda and co-workers have reported the synthesis of a 5-acetyl-2-aminopyrrole nucleoside 22.^89^ Direct coupling of the initial base analogue 15*via* Heck-coupling was unsuccessful. The authors attributed this limitation to the diminished oxidative addition and palladium insertion associated with strongly electron donating aromatic systems. To overcome this challenge, the corresponding nitro-substituted aryl precursor 18 was employed. In this case, the coupled intermediate could be obtained and later reduced to the desired amino compound as outlined in Fig. 5.^89^ One application of the pyrrole base analogue lies in reverse transcription of RNA into cDNA. The modified H-bonding pattern of the analogue would make it for example suitable to pair with 2,2,7-trimethyl-G (23), a natural RNA modification that could thus be detected by sequencing. In the same year, Oda and co-workers also described a complementary triazine based nucleoside analogue 24 prepared *via* the Heck-coupling reaction (Fig. 5).^90^ Hocek reported a related observation in the context of Heck-coupling to furanoid glycals. Direct coupling of the electron poor 3-iodo-6-chloropyridone was not observed, but when the pyridone was transformed into a phosphorodiamidate, the compound coupled smoothly in high yield (73% incl. desilylation).^91^

McLaughlin and co-workers have employed unnatural C-nucleosides as chemical probes to investigate structure–function relationships in catalytic nucleic acids. For example the hammerhead ribozyme has been investigated by substituting a defined uridine residue with a C-glycosidic nitropyridone analogue and monitoring the resulting changes in cleavage kinetics.^92^ Similarly alternatively modified nucleosides have been incorporated, including a C-glycosidic aminopyridine.^93^ Romesberg, who later investigated an exhaustive library of nucleobase analogues to find efficient orthogonal base pairs, used the Heck reaction to synthesize a library of benzene nucleosides, predominantly containing methyl and methoxy substituents.^50^

Examples of the obtained nucleoside analogues were later used in the optimisation of an unnatural base pair for enzymatic replication.^94^ Similar methyl- and methylaminobenzenes were also reported by Heo and Hwang *via* Heck-coupling.^95^ Romesberg and co-workers observed that Heck-coupling of amino-methoxy benzene (AMO1, Fig. 7) required Cbz protection of the amine to lower electron density of the aromatic system for sufficient yields. After deprotection to the free amine, the nucleoside was derivatised to generate nitro-, N-pyrrole, or (trifluoro)acetamide variants. The impact of these substituents on incorporation kinetics was systematically investigated in primer extension assays using Taq DNA polymerase opposite the hydrophobic nucleobase analogue 5SICS.^96^

Within the realm of unnatural base pairs, one of the most extensively studied systems is the TPT3–NaM pair. TPT3, in its N-glycosidic form, suffers from being susceptible to acidic or enzymatic cleavage of the glycosidic bond. Kath-Schorr and co-workers addressed this limitation by developing the C-glycosidic analogue CTPT3 (Fig. 7), which could be efficiently incorporated opposite NaM in both primer extension and *in vitro* transcription reactions.^18^ The corresponding 2′-deoxy-nucleoside dCTPT3 was prepared *via* a Heck reaction employing 15 mol% Pd(dppf)Cl_2_, representing one of the few reported examples of an unnatural base pair based on C-glycosides introduced enzymatically using a Heck-derived scaffold.

**Fig. 6:**
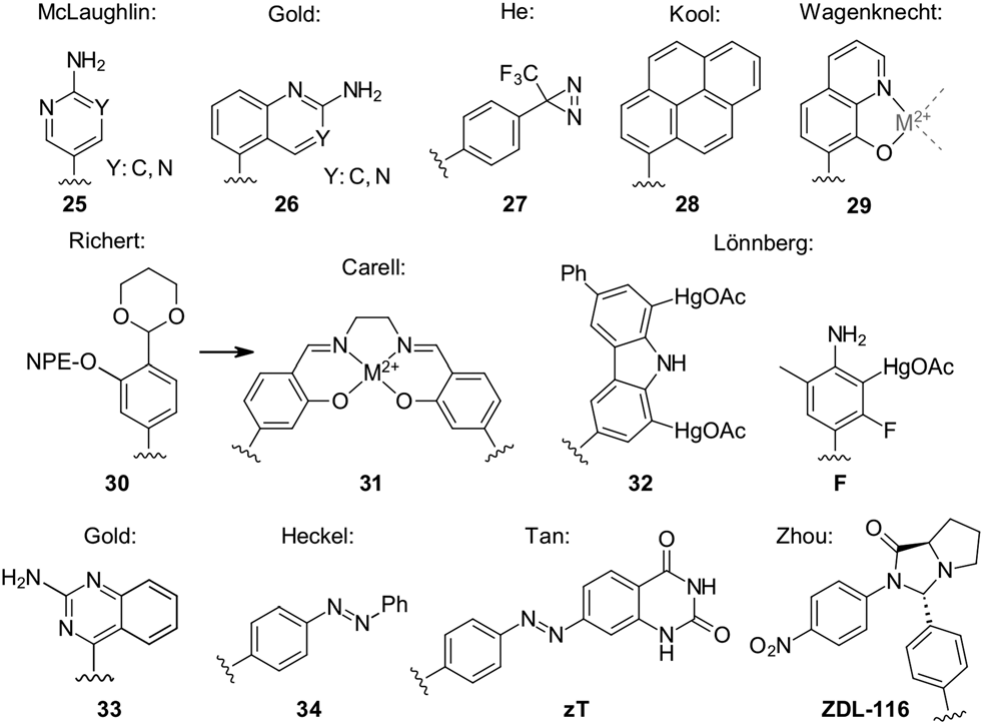
C-nucleosides with special modes of action, including triplex-inducing, inter-strand cross-linking, or metal-coordinating ones.

A systematic evaluation of reaction parameters for the Heck-coupling of 2-chloropyridines was described by Hocek and co-workers.^63^ They reported the reaction to only efficiently work in chloroform and that a key component for reactivity is the use of triphenylarsine as a ligand and silver carbonate as base. In addition, they highlight the higher reactivity of 5′-deprotected glycals as an important factor for successful coupling. The obtained chloropyridinyl-nucleoside was then further derivatised by replacing chlorine with other substituents by amination or Pd-catalysed cross-coupling, thereby enabling further structural diversification. He expanded the substrate scope of Heck derived C-nucleosides by introducing a diazirine moiety (27, Fig. 6).^97^

**Fig. 7:**
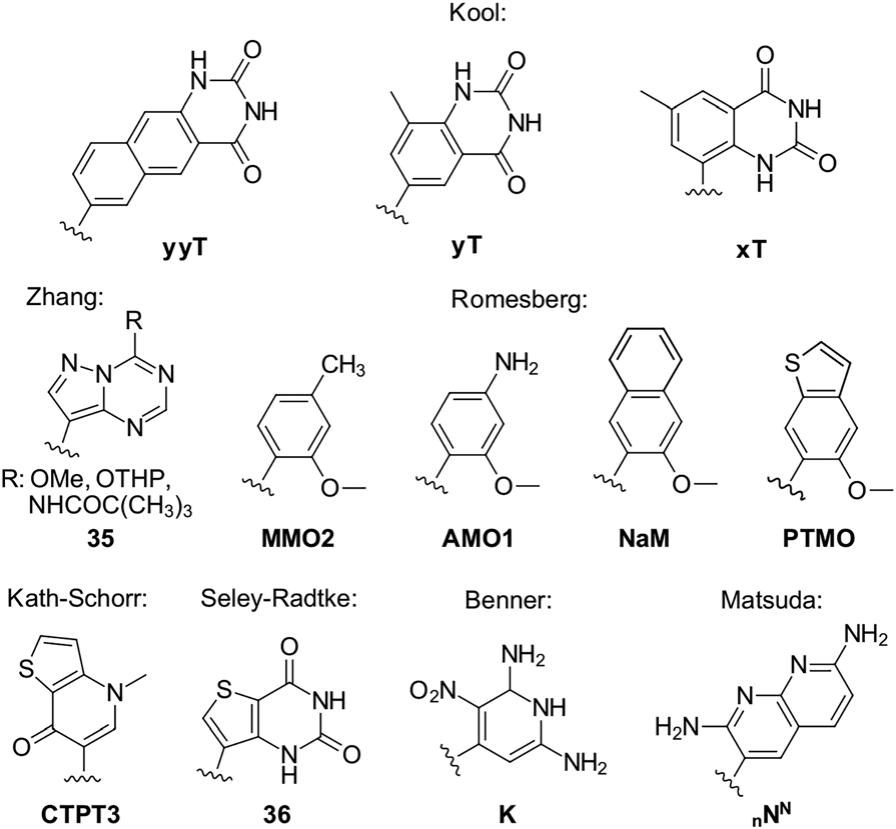
Enlarged nucleobase analogues, C-nucleosides used in unnatural base pairs and a four-fold hydrogen-base-pairing analogue.

The corresponding nucleosides were converted into phosphoramidite building blocks and successfully incorporated into oligonucleotides bearing the diazirine functionality. Upon photolysis, generation of a reactive carbene intermediate enabled covalent cross-linking within double-stranded DNA. This photoactivatable reactivity suggests potential applications as a probe for studying NA-NA or NA-protein interactions. In addition, the authors proposed that such constructs may hold promise as photochemically triggered agents in anticancer strategies.

Hou and Greenberg have synthesized a series of haloaromatic C-nucleosides, incorporated them into oligonucleotides by solid-phase synthesis and investigated their radical-mediated inter-strand cross-linking behaviour *via* photolysis of the X–Ar bond.^98^

The authors proposed potential applications of these constructs in photochemical cancer therapy. However, the corresponding nucleoside triphosphates were not accepted as substrates by Deep Vent (*exo*-) DNA polymerase, limiting their utility in enzymatic incorporation strategies.

Kool employed fluorogenic nucleobase analogues, including C-nucleosidic pyrene derivatives, to investigate base excision repair, the cellular pathway responsible for processing abasic sites generated by spontaneous depurination or enzymatic removal of damaged bases. In specially designed triplex templates, the sterically demanding pyrene base selectively binds at abasic sites. This binding event displaces a proximal quencher, resulting in turn-on fluorescence and enabling sensitive detection of repair relevant intermediates.^99^

Gold and co-workers made a 2-amino-quinazoline nucleobase analogue 26 that promotes the formation of nucleic acid triplex structures.^100^ Incorporation of this C-nucleosidic analogue was shown to stabilize triplex assemblies, highlighting the capacity of expanded heterocycles to modulate higher order DNA architectures.

Heckel and co-workers expanded the scope of the Heck reaction by introducing diazobenzene motifs, reporting the synthesis of a C-nucleosidic photoswitch 34 capable of modulating RNA hybridisation upon irradiation.^101^ Photoisomerization of the azobenzene unit altered duplex stability, thereby enabling light controlled regulation of RNA structure. Expanding on this concept Tan and co-workers developed a diazobenzene-expanded thymine analogue *zT*, which they incorporated into DNA and forms base pairs with adenines that can be disrupted by UV-irradiation (Fig. 6).^102^

### Metal-binding nucleosides

Richert and co-workers revised the synthesis of a salicylic acid nucleoside 30 by implementing a the Heck-coupling strategy.^103^ The resulting nucleoside was converted into a phosphoramidite building block suitable for solid phase synthesis. Oligonucleotides containing this ligand-bearing nucleotide enable cross strand chelation of metal ions, consistent with earlier concepts introduced by Carell and colleagues (31).^104^

Wagenknecht and co-workers have used 8-hydroxy-quinolines (29) as nucleobase analogues to generate metal binding sites within DNA duplexes. Oligonucleotides prepared by solid phase synthesis were examined in the presence and absence of copper ions, and comparison of melting temperatures revealed metal dependent stabilization effects indicating coordination within the duplex.^105^ Electron paramagnetic resonance (EPR) measurements were performed, to probe the metal–metal interactions in the assembled systems. In addition, constructs were designed in which differently long stretches of the hydroxyquinoline base pairs were placed in between a charge-donor- and charge-acceptor-modified nucleotide to investigate their charge-transfer behaviour.

The working group of Lönnberg has used the Heck reaction to synthesize different C-nucleosides designed for metal coordination within duplex DNA. In 2019 they reported a 3-fluoro-6-methylaniline nucleobase analogue F (Fig. 6), which can be mercurated in 2-position^106^ and later a similar structure containing a CF_3_-group as sensitive probes to investigate the mercury-mediated base pairing *via* NMR.^107^ When incorporated into duplexes, analysis of melting temperatures together with fluorine chemical shifts permitted discrimination between different natural nucleotides located opposite the modified base. These systems thus function as spectroscopic probes for metal mediated pairing interactions. In addition, the group described a carbazole derived nucleoside analogue (32) capable of binding two mercury atoms, further expanding the repertoire for controlled metal coordination within nucleic acid architectures.^108^

Starting from the 2-bromopyridin-5-yl coupling product *via* Heck reaction^63^ Lönnberg recently synthesized expanded pyrenyl-pyridine nucleosides, which could be cyclopalladated within ssDNA. This metalation retarded duplex formation which indicates non-natural duplex geometry, but the detected fluorescence was dependent on the identity of the opposing base, indicating potential utility of these cyclopalladated C-nucleosides as base discriminating sensing-elements.^70^

Kool and co-workers synthesized different nucleosides bearing fluorescent or metal binding functionalities and immobilized corresponding oligonucleotide sequences on polystyrene beads to generate sensor platforms for metal contaminants in water.

Fluorescence measurements enabled identification and quantification of different contaminant species.^109^

Zhou coupled *p*-iodo-benzaldehyde *via* Heck-coupling on the way to the antiviral nucleoside candidate ZDL-116.^110^ This example illustrates the continued relevance of C-glycosidic bond formation in medicinal nucleoside development.

### Unnatural nucleobase pairs

The Kool group developed systematic sets of nucleobase analogues designed to mimic the hydrogen bonding patterns of natural bases while increasing the inter-strand sugar-to-sugar distance within the duplex. Two principal classes were introduced. The expanded *x*^111^ and the widened *y*^112^ series, both incorporate benzene rings into the natural nucleobase structures, although at different attachment angles (Fig. 7). Subsequent work extended this concept to even larger naphthyl expanded nucleobases such as yyT.^113^

Seley-Radtke reported the synthesis of thieno-extended pyrimidine C-nucleosides 36 and investigated their anti-proliferative effects on certain cancer cell-lines,^114^ which shows the potential of structural expansion of the nucleobase scaffold for therapeutic applications. Matsuda and co-workers have described the design and synthesis of base analogues forming four hydrogen bonds per base pair (_n_N^N^). The C-glycosidic representatives of these systems were prepared *via* the Heck reaction, providing access to artificial base pairs with enhanced hydrogen bonding capacity and altered duplex properties.^115^ Benner employed the Heck reaction to synthesize several C-nucleosides that complement the artificially expanded genetic information system (AEGIS). This system is composed of non-canonical base analogues featuring rearranged hydrogen bonding patterns (K), enabling the formation of orthogonal base pairs beyond the natural genetic alphabet.^116,117^

## Conclusions

In summary, the Heck reaction has established itself as a central transformation in nucleic acid chemistry. The development of furanoid glycals as stable and versatile precursors for C–C bond formation, combined with stereochemical control imparted by protecting group strategies, has enabled efficient and often highly selective access to C-glycosidic nucleosides. A key advantage of this approach is the inherent preference for formation of the β-anomeric configuration, which directly addresses one of the most critical selectivity challenges in C-glycoside synthesis and is a major reason for its widespread adoption. Particularly for suitably activated electrophiles, robust coupling yields have been achieved, rendering this methodology highly practical for synthetic applications.

Over the past decades, numerous research groups have exploited this reaction to construct artificial C-glycosides, motivated either by the enhanced stability of the carbon–carbon linkage relative to N-glycosides or by the structural demands of unnatural base pair engineering. While other methods lack broader substituent tolerance and synthetic reliability, these works contributed to the great success of this cross-coupling reaction in nucleic acid chemistry.

Furthermore, many of the DNA nucleosides have been implemented in diverse applications, from precursors for late-stage functionalisation to unnatural base pairs and intrinsically fluorescent nucleosides.

Despite these advances, additional systematic investigations of Heck reactions with furanoid glycals are warranted. In particular, a comprehensive exploration of the substrate scope and detailed evaluation of palladium catalyst ligand combinations are required to define generally applicable conditions. While the current state-of-the-art system is Pd(OAc)_2_/AsPh_3_, selected substrates clearly benefit from tailored catalytic complexes. Overall, the Heck reaction represents a powerful and enduring tool for the synthesis of artificial nucleic acids. Its characteristic β-selectivity, in combination with its robustness and functional group tolerance, makes it one of the most attractive strategies for accessing C-glycosidic nucleosides and underpins its central role in nucleic acid chemistry. Its continued refinement will facilitate access to increasingly complex C-glycosides and thereby further expand the chemical diversity available to engineered genetic systems.

## Conflicts of interest

There are no conflicts to declare.

## Data Availability

All figures were prepared by the authors, and no additional external data were used in this review.
